# Microbiome‐Driven Mechanisms in Breast Cancer: Emerging Evidence From Gut Microbial Signatures to Therapeutic Response

**DOI:** 10.1155/bmri/8376859

**Published:** 2026-08-11

**Authors:** Adil Farooq Wali, Sirajunisa Talath, Imran Rashid Rangraze, Mohamed El-Tanani, Shehla Khan

**Affiliations:** ^1^ RAK College of Pharmacy, RAK Medical and Health Sciences University, Ras Al Khaimah, UAE; ^2^ RAK College of Medical Sciences, RAK Medical and Health Sciences University, Ras Al Khaimah, UAE

**Keywords:** breast cancer, dysbiosis, estrobolome, gut microbiome, immunotherapy, microbial metabolites

## Abstract

Breast cancer remains the most frequently diagnosed malignancy among women worldwide, and increasing evidence indicates that the gut microbiome plays a significant role in tumor initiation, progression, and therapeutic response. Microbial dysbiosis and altered metabolite production have been associated with systemic inflammation, estrogen metabolism, immune regulation, and metabolic reprogramming, all of which contribute to breast cancer biology. This review summarizes current preclinical and clinical evidence describing the gut–breast cancer axis and its mechanistic and translational relevance. The review focuses on four major pathways through which gut microbiota may influence breast cancer development and treatment outcomes: immune modulation, estrobolome‐mediated estrogen recycling, chronic inflammatory signaling, and microbial metabolite‐driven epigenetic and metabolic regulation. Evidence from experimental models and human studies demonstrates that alterations in microbial diversity and enrichment of proinflammatory taxa are associated with tumor progression, subtype‐specific biology, and variability in therapeutic response. Emerging findings further indicate that microbiome composition can influence the efficacy and toxicity of chemotherapy, endocrine therapy, radiotherapy, and immunotherapy, highlighting the potential of microbiome‐informed precision oncology strategies. In addition, this review discusses current advances in microbiome‐targeted interventions including probiotics, dietary modulation, postbiotics, and fecal microbiota transplantation. Despite promising translational potential, significant challenges remain regarding mechanistic validation, standardization of microbiome profiling, reproducibility across cohorts, and clinical implementation. Future research integrating longitudinal multiomics approaches, functional validation studies, and personalized microbiome‐based therapeutic strategies may facilitate the development of clinically actionable microbiome interventions for breast cancer management.

## 1. Introduction

Breast cancer (BC) is the most common cancer in women worldwide and continues to be associated with considerable morbidity and mortality despite early detection and advances in therapy [1]. According to the latest GLOBOCAN estimates reported by the World Health Organization (WHO) and the International Agency for Research on Cancer (IARC), BC remains the most commonly diagnosed malignancy among women worldwide, accounting for approximately 2.3 million new cases and 685,000 deaths globally. Current projections further indicate that the global burden of BC may increase to nearly 3 million new cases annually by 2040 due to population growth and aging. These alarming trends highlight the urgent need for improved strategies to prevent and treat BC through early detection, precision medicine, and novel therapeutic approaches. [2]. The pathogenesis of BC has been traditionally investigated in the context of genetic susceptibility, hormonal control and environmental exposures [3]. Emerging data from studies utilizing high‐throughput sequencing, metagenomics and metabolomics have unveiled unique microbial signatures present in BC patients that warrant further investigation as potential biomarkers for risk of disease, tumor subtype and clinical outcome [4]. Nonetheless, a growing body of evidence points to the gut microbiome as an important but understudied player in BC initiation and progression, generating the idea of a gut–BC axis. This axis reflects a complex, bidirectional crosstalk wherein gut microbiome affects systemic metabolism, immune regulation and hormonal environment, which are directly interlinked with breast tissue homeostasis and cancer development [5].

There are trillions of microorganisms in the human gastrointestinal system including bacteria, viruses, fungi, and archaea as gut microbiota. These microbes undergo a constant crosstalk with the host and secrete metabolites like short‐chain fatty acids (SCFAs), bile acids, and tryptophan derivatives that have been linked with systemic inflammatory responses, estrogen metabolism, and oncogenic signaling pathways [6, 7]. All preclinical and clinical studies indicate that the microbiome composition can influence response to  chemotherapy, immunotherapy, and hormone therapy. Individual taxa may increase drug metabolism, change immune checkpoint function, or reduce the toxicities of treatment, categorizing the gut microbiome as both a predictive biomarker and a target for therapy [8].

Altogether, the microbiome is in a central position as a key modulator of BC biology, merging signals from remote organs, from local tissue niches, and transversal metabolic and immune systemic networks. The frameworks allow us to consider microbial composition as a disease risk biomarker and an intervenable axis, with the potential to modify tumor biology through diet, probiotics, prebiotics, and microbiota‐directed pharmacologic strategies [9, 10].

To provide a unifying framework for the evidence discussed in this review, we propose a central conceptual model in which gut and breast‐associated microbiomes influence BC initiation, progression, and treatment response through a limited set of core host pathways. Perturbation of these host pathways collectively shapes downstream clinical endpoints, including BC risk, tumor subtype–specific biology, disease progression, therapeutic efficacy, treatment‐related toxicity, and patient prognosis. This integrative microbiome–host–clinical axis provides a conceptual scaffold for organizing mechanistic insights and translational implications discussed throughout the manuscript.

This review synthesizes evidence showing that microbiome‐regulated estrogen metabolism intersects with immune modulation to shape BC progression and response to endocrine therapy and immunotherapy, moving beyond models that consider hormonal or immune mechanisms in isolation.

### 1.1. Search Strategy and Evidence Selection

This narrative review was conducted through a comprehensive literature search of peer‐reviewed studies investigating the relationship between the gut microbiome and BC biology, progression, and therapeutic response. Electronic databases including PubMed, Scopus, Web of Science, and Google Scholar were searched for studies published between January 2010 and March 2026. The search strategy used combinations of keywords such as “breast cancer,” “gut microbiome,” “microbiota,” “dysbiosis,” “estrobolome,” “microbial metabolites,” “immunotherapy,” “endocrine therapy,” and “precision oncology.”

Both preclinical and clinical studies were considered for inclusion. Original research articles, translational studies, clinical trials, and high‐quality review articles published in English were included based on their relevance to microbiome‐mediated mechanisms in BC, including immune modulation, estrogen metabolism, inflammatory signaling, metabolic reprogramming, and therapeutic response. Editorials, duplicate studies, conference abstracts lacking sufficient methodological details, and studies not directly related to BC–microbiome interactions were excluded.

The evidence included in this review was synthesized qualitatively with emphasis placed on mechanistic consistency, translational significance, and emerging clinical applicability. Priority was given to recent studies employing metagenomics, metabolomics, multiomics integration, and longitudinal clinical designs to provide an updated and comprehensive overview of the field.

## 2. Gut Microbiome Architecture and Functional Dynamics

### 2.1. Composition and Diversity of the Gut Microbiome

The gut microbiome is an extremely complex and dynamic ecosystem consisting of trillions of microorganisms, including bacteria, archaea, viruses, fungi, and protozoa that together affect host physiology as well as susceptibility to diseases [11]. Within the context of BC, insight into the makeup and diversity of microbes is crucial to understanding how gut bacteriome influences systemic and local tumor biology. The gut ecosystem is colonized by bacteria that comprise most of its population, which are mostly members of the phyla Firmicutes, Bacteroidetes, Actinobacteria, and Proteobacteria, contributing unique metabolic and immunomodulatory activities [12].

The diversity between microorganisms includes richness and evenness (the quantity of unique taxa and the uniformity of their distribution), which also have a great influence on gut ecosystem′s stability and functional resilience [13]. A rich microbial diversity is generally luxurious and associated with increased metabolic flexibility, immune competence, and resistance to pathogenic colonization, whereas low diversity types like dysbiosis have been reported elsewhere, and it is correlated with systemic inflammation, altered metabolism/immunology of estrogen metabolism, then decreased that can be a risk factor for BC development. In BC populations, analysis has also observed a tendency to reduce alpha diversity and modification of some taxa associated with tumor subtype, clinicopathological stage, and response to therapeutics. For example, increased levels of proinflammatory bacteria (e.g., *Escherichia coli* and *Enterococcus* species) on the one hand, and reduction of beneficial commensals (*Bifidobacterium* and *Lactobacillus*), have been documented, indicating that changes in microbial composition may create a protumorigenic systemic environment [14].

### 2.2. Microbial Homeostasis and Dysbiosis

Microbial homeostasis refers to the balanced coexistence between the host and the diverse microbial communities inhabiting the gastrointestinal tract. Under physiological conditions, the gut microbiome contributes to nutrient metabolism, maintenance of epithelial barrier integrity, immune maturation, protection against pathogenic colonization, and regulation of systemic inflammatory responses. A stable microbial ecosystem is generally characterized by high microbial diversity, functional redundancy, and dynamic equilibrium among beneficial commensal microorganisms. Dominant bacterial phyla such as Firmicutes, Bacteroidetes, Actinobacteria, and Proteobacteria collectively participate in maintaining intestinal and systemic homeostasis through the production of bioactive metabolites, modulation of immune pathways, and preservation of mucosal integrity.

The gut microbiota also plays a fundamental role in regulating host metabolism and immune surveillance through the synthesis of SCFAs, bile acid metabolites, vitamins, and other microbial‐derived signaling molecules. Metabolites such as butyrate, acetate, and propionate contribute to epithelial barrier maintenance, anti‐inflammatory signaling, and regulation of T cell differentiation. In addition, commensal microorganisms influence endocrine and neuroimmune pathways, thereby affecting distant organs beyond the gastrointestinal tract, including the liver, brain, and breast tissue. Through continuous bidirectional communication with host immune and metabolic systems, the microbiome acts as a central regulator of systemic physiological equilibrium.

Dysbiosis refers to a disruption in the composition, diversity, or functional activity of the gut microbiome that may contribute to pathological processes. This imbalance can manifest as reduced microbial diversity, loss of beneficial commensals, overgrowth of opportunistic pathogens, or altered microbial metabolic activity. Multiple factors including diet, antibiotic exposure, aging, obesity, environmental toxins, chronic stress, infections, hormonal fluctuations, and lifestyle changes can promote dysbiosis. Emerging evidence suggests that persistent microbial imbalance contributes to chronic low‐grade inflammation, immune dysregulation, metabolic dysfunction, and aberrant signaling pathways associated with carcinogenesis.

In the context of cancer biology, dysbiosis has been implicated in promoting tumor initiation and progression through several interconnected mechanisms. Altered microbial communities may enhance inflammatory cytokine production, increase oxidative stress, disrupt epithelial barrier integrity, and influence estrogen metabolism through microbial *β*‐glucuronidase activity. Dysbiotic microbiota can also modulate immune checkpoint pathways, antigen presentation, and tumor‐associated immune responses, thereby influencing both tumor progression and therapeutic responsiveness. In BC specifically, several studies have reported altered gut microbial diversity and enrichment of proinflammatory bacterial taxa associated with disease progression, although many findings remain associative and require further mechanistic validation.

Despite increasing interest in microbiome‐associated carcinogenesis, interpretation of microbial alterations should be approached cautiously because microbiome composition is highly dynamic and influenced by multiple host and environmental variables. Furthermore, variations in sampling methods, sequencing platforms, analytical pipelines, and cohort characteristics contribute to heterogeneity across studies. Understanding the balance between microbial homeostasis and dysbiosis is therefore essential for elucidating the role of the microbiome in BC biology and for developing future microbiome‐targeted therapeutic strategies.

### 2.3. Host–Microbiome Interactions in Health and Disease

The interaction between host and microbiome also plays a key role in the capacity for the gut microbiota to heavily influence carcinogenesis, including BC, through systemic immune responses, metabolic signaling, hormonal balance, and inflammatory pathways [15, 16]. At the mucosal surface, pattern recognition receptors such as toll‐like receptors (TLRs), nucleotide‐binding oligomerization domain (NOD)‐like receptors, and C‐type lectin receptors recognize microbial‐associated molecular products (MAMPs) including lipopolysaccharides (LPS), peptidoglycans, and flagellin [17]. These receptors initiate signaling cascades that can be expected to impact cytokine production, recruitment of immune cells, and integrity of the epithelial barrier, thereby being capable of changing systemic inflammation and even conditions promoting tumor formation.

The host–microbiome interactions and their immune modulatory role are also related to BC development. Gut microbes influence both innate and adaptive immunity through their effects on the differentiation and activation of dendritic cells (DCs), macrophages, natural killer (NK) cells, and T lymphocytes [18]. Some of the commensal bacteria like *Bifidobacterium* and *Akkermansia* have been associated with improved antitumor immunity through cytotoxic T cell infiltration, DC maturation, and modulation of regulatory T cell subset [19].

**Figure 1:**
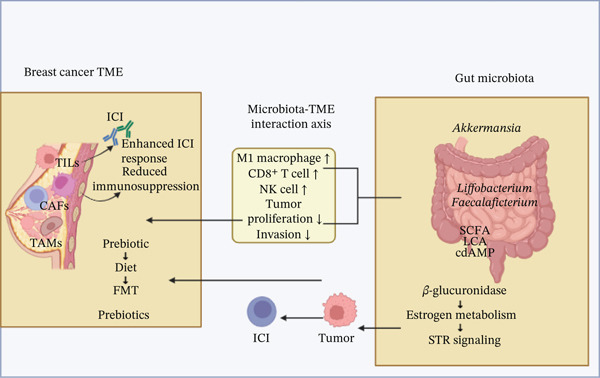
Microbiota‐driven remodeling of breast cancer TME enhances immunotherapy response. This figure shows potential mechanisms through which gut microbiota modulation can improve breast cancer immunotherapy through remodeling of the tumor microenvironment (TME). An overview of breast cancer TME on angle breast cancer TME with TILs, CAFs, and TAM. ICIs work by enhancing antitumor immune responses and dampening immunosuppression. This environment is affected by interventions including prebiotics, dietary modulation and fecal microbiota transplantation (FMT). The microbiota–TME interaction route indicates the favorable immunological effects by enhanced M1 macrophages, CD8 + T‐suppression and NK, which proximately inhibit the tumor cell proliferation and invasion as shown in the middle. The image on the right shows some gut microbes that are producers of metabolites (e.g., *Akkermansia*, *Lactobacillus*, and *Faecalibacterium*) such as SCFAs, lithocholic acid (LCA), and cyclic diadenosine monophosphate (cdAMP). These metabolites modulate *β*‐glucuronidase activity, hormone metabolism and signaling, with downstream effects on immune activation that attenuate tumor progression. Collectively, the figure underscores the therapeutic promise of harnessing gut microbiota modulation in enhancing immunotherapy for breast cancer.

Recent findings reinforce the role of microbial metabolites as an additional dimension of host–microbiome cross talk in cancer biology, with implications for epigenetic regulation. SCFAs such as butyrate are inhibitors of histone deacetylases and they modulate chromatin accessibility and gene expression in immune cells and epithelial cells [20]. Metabolites from the tryptophan catabolism into indoles are also a signal for aryl hydrocarbon receptor (AhR) signaling pathway, which guides immune tolerance, inflammation, and epithelial homeostasis. Such changes modulating expression of the oncogenes, tumor suppressor genes, and those involved in DNA repair could potentially be integrated into some fundamental aspects of BC tumorigenesis [21]. Figure 1 shows microbiota‐driven remodeling of BC during TME enhances immunotherapy response.

### 2.4. Breast Tissue Microbiota

Molecular subtype and hormone receptor (HR) status allow for stratification of the microbiota in breast tumors [22]. Tumors that tested positive for HR and those that tested negative showed notable microbiological differences. *Paracoccus*, *Actinomyces*, *Hydrogenophaga*, *Halomonas*, *Cutibacterium granulosum*, *Bacillus cereus*, *Staphylococcus aureus*, *Clostridium tetani*, *Acinetobacter baumannii*, and *Spirosoma pollinicola* are among the bacteria that are more abundant in HR‐positive samples [23]. It is possible that the translocation or colonization dynamics unique to HR‐positive tumor microenvironments (TMEs) are mirrored by the fact that many of these genera are skin‐associated or ambient microorganisms. On the other hand, HR‐negative tumors may affect the inflammatory environment due to elevated numbers of *Acinetobacter*, *Rhodobacter*, *Streptomyces*, *Burkholderiaceae*, and *Priestia megaterium* [23]. *Alkanindiges*, *Micrococcus*, *Caulobacter*, *Proteus, Brevibacillus, Kocuria,* and *Parasedimini bacterium* were found in significantly lower abundances in ER‐positive tumors as compared with ER‐negative tumors, according to another study [24]. This group of organisms includes both commensal and opportunistic species. Molecular subtype and hormone receptor (HR) status allow for stratification of the microbiota in breast tumors, revealing distinct microbial profiles associated with different breast cancer subtypes and receptor statuses [25].

New research has linked certain bacterial genera to PgR+ cancers as well. *Pelomonas, Ralstonia*, *Oblitimonas*, *Lactobacillus*, *Methylophilus*, and *Achromobacter* were found in greater abundance in PgR+ tumors [24]. Furthermore, the unique microbiome compositions linked to tumor subtypes have been highlighted by the strong clustering of microbial profiles based on progesterone receptor status that was detected using Bray–Curtis dissimilarity (*p* = 0.044). The immunological interactions specific to the human epidermal growth factor 2 (HER2)‐TME may be reflected in the elevated abundances of *Cloacibacterium*, PRD01a011B, Al‐loprevotella, *Stakelama*, *Filibacter*, *Blastomonas*, and *Anaerostipes* observed in HER2‐tumors [24].

## 3. Breast Tissue Microbiome and TME

### 3.1. Altered Gut Microbial Signatures in Breast Cancer Patients

Recent research has strongly suggested that BC is not a local disease but also affected by systemic factors, such as the gut microbiota composition and their biological effects [26]. Both qualitative and quantitative alterations in bacterial taxa have been reproducibly observed by high‐throughput sequence analysis (16S rRNA gene profiling, metagenomics). For instance, patients with BCP also show reduced abundance of beneficial commensals *Lactobacillus*, *Bifidobacterium*, *and Faecalibacterium prausnitzii*, which possess anti‐inflammatory and immunomodulatory activities [27]. Conversely, an abundance of potentially pathogenic or proinflammatory taxa such as *Escherichia*/*Shigella*, *Enterococcus*, and specific members of the Proteobacteria have been described, implying a skewed microbial community that could promote systemic inflammation (a mechanism known to facilitate oxidative stress and disregulate estrogen metabolism and implicated in breast carcinogenesis) [28]. Patterns of bacterial dysbiosis also appear to differ by tumor subtype, receptor status, and stage. Patients with hormone receptor‐positive (HR+) tumors, for example, frequently have a unique gut microbiome signature that includes enrichment of bacteria that can influence estrogen metabolism through the estrobolome: microbial genes responsible for deconjugation of estrogens. It causes increased circulating estrogen that further stimulates the growth of hormone‐sensitive BCs [29].

### 3.2. Microbial Metabolite Shifts

SCFAs such as acetate, propionate, and butyrate are mainly derived from fermentation of dietary fibers by commensal bacteria like *F. prausnitzii*, *Roseburia* spp., and *Bifidobacterium* spp. [30]. These SCFAs have multifaceted effects on host physiology, such as immunomodulation by balancing host immunity, epigenetic regulation through inhibition of histone deacetylases, and aiding in the maintenance of gut barrier function. In BC, imbalances in those chains of SCFA bacteria were related regressively to systemic inflammation, immune monitoring disorder, and proliferative signaling [31].

In addition to SCFAs, another crucial microbial‐associated pathway that also affects breast carcinogenesis is bile acid metabolism. The gut bacteria that promote the primary‐to‐secondary bile acid conversion and subsequent metabolites such as deoxycholic acid (DCA) and lithocholic acid (LCA) may act as signaling molecules via nuclear receptors such as FXR or TGR5, causing an influence on lipid metabolism, estrogen receptor (ER) activity, and tumor‐promoting local microenvironments [32]. Dysbiosis‐associated changes in bile acid composition have been associated with elevated levels of oxidative stress, DNA damage, and procarcinogenetic cytokine release that collectively provide an environment favorable for tumorigenesis [33]. Table 1 showing estrobolome‐immune‐therapy convergence as microbiome‐driven pathways for BC biology.

**Table 1:** Core microbiome‐driven pathways influencing breast cancer biology.

Pathway category	Key biological mechanism	Functional consequence in breast cancer	Translational relevance
Immune modulation	PRR signaling (TLRs/NLRs), antigen presentation, CD8^+^ T cell activation	Shapes tumor immune microenvironment; influences immune surveillance and immune evasion	Predictive of immunotherapy response
Estrogen regulation (estrobolome)	Microbial *β*‐glucuronidase–mediated estrogen recycling	Alters systemic and local estrogen exposure, particularly in ER^+^ disease	Modulates endocrine therapy efficacy
Inflammatory signaling	NF‐*κ*B and IL‐6/STAT3 pathway activation	Promotes chronic inflammation, EMT, and angiogenesis	Targetable via microbiome modulation
Metabolic reprogramming	SCFAs and bile acid signaling (FXR/TGR5)	Epigenetic regulation, metabolic plasticity, and immune tone	Diet‐ and metabolite‐based interventions
Epigenetic control	HDAC inhibition, DNA methylation modulation	Tumor suppressor activation, reduced proliferation	Supports postbiotic strategies

### 3.3. Microbiome Changes Across Breast Cancer Subtypes

Three common BC subtypes—HR+, overexpressing HER2 (HER2‐enriched) and negative for both ER/PR and HER2 when tested (triple‐negative, TNBC) show unique microbial signatures representing the varying immune modulation, metabolism, and local microenvironment interactions [34]. Enrichment of microbiota functions for estrogen metabolism has also been found in ER‐positive tumors, a functionally active estrobolome with the capability of regulating systemic and local estrogen levels that lead to the promotion of tumor proliferation by members of Clostridiales and Bacteroidales orders [35–37].

Functional metagenomic analyses demonstrate that the subtype‐specific microbial signatures are associated with distinct metabolome profiles, including SCFAs, secondary bile acids, and estrogen metabolites capable of exerting impact on the epigenetic modifications, immune cell migration, and hormone signaling in a subtype‐dependent manner [38]. Critically, these microbiome signatures have relevance in the clinic and could impact responses to endocrine therapy, HER2‐targeted agents, chemo/radiation, and next‐generation immunotherapies, raising the possibility that incorporating microbial profiling into BC stratification might deliver precision interventions [39].

### 3.4. Mechanisms of Microbiome Influence on BC

**Figure 2:**
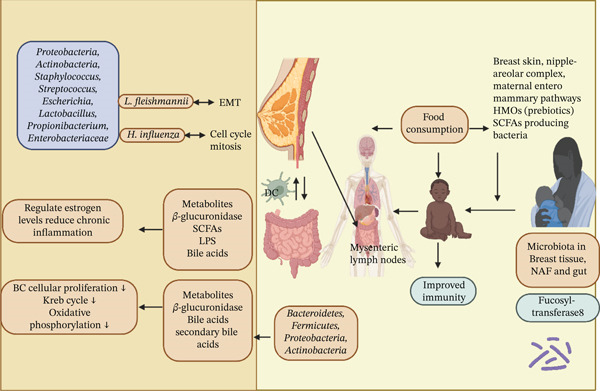
Microbiome‐mediated modulation of the breast tumor microenvironment. In the figure, it shows how microbiota from breast tissue, nipple–areolar complex, gut and breast milk affect breast cancer development and infant immunity. Tumor growth is modulated by specific bacteria and their metabolites (*β*‐glucuronidase, SCFAs, bile acids, and LPS) that regulate estrogen metabolism, inflammation, and cellular energy pathways. Simultaneously, maternal entero‐mammary transfer with human milk oligosaccharides (HMOs) promotes beneficial SCFA‐producing bacteria, thus supporting immune maturation in the neonate. In summary, the figure illustrates multiple sites where microbiota can either modulate BC biology and/or enhance the pick of immunity by infants.

Numerous bacterial species have been identified in studies, both animal and human, of BC tissues. It has been shown that bacteria such as *Staphylococcus* and *E. coli* could induce DNA DSBs and genomic instability in vitro. It may potentially contribute to carcinogenesis in human tissues [40]. Interestingly, certain Clostridiales can inhibit tumor growth, by promoting antitumor CD8+ T cell responses dependent on trimethylamine N‐oxide (TMAO) production [41]. It has been reported that the *Fusobacterium nucleatum* can adhere to Gal‐GalNac in Fap‐2 dependent fashion of BC tissue, which promotes tumor growth and metastasis [42]. Via modulating the stress response and modifying cancer cell viability as well as cytoskeletal components, species of *Staphylococcus*, *Lactobacillus* spp., and *Streptococcus* have been associated with lung metastases from breast tumors in mice [43]. *S. epidermidis* is also thought to induce complement and enhance T regulatory cells infiltrating in the tumor [26]. Although, *M. luteus* is a candidate antitumor agent for their suppressive effect on mammary tumor growth and for promoting an antitumoral macrophage M1 phenotype, *Bacteroides fragilis* has been associated to the proliferation and invasion of breast tumors (BFT: *Bacteroides fragilis* toxin) [26]. Figure 2 shows microbiota across breast, gut, and milk regulate BC progression and infant immunity.

Schematic illustration demonstrating the influence of gut microbial dysbiosis on the breast TME. The figure highlights microbiome‐associated regulation of immune signaling, inflammatory cytokine production, estrogen metabolism, epithelial barrier dysfunction, and microbial metabolite activity contributing to tumor progression. Dysbiotic microbial communities may influence CD8+ T cell activity, regulatory T cell responses, macrophage polarization, angiogenesis, and metastatic signaling pathways within the BC microenvironment. The diagram also illustrates the role of microbial metabolites such as SCFAs and secondary bile acids in immune modulation and epigenetic regulation associated with BC progression.

## 4. Estrobolome and Mechanistic Pathways in BC

### 4.1. Estrogen Metabolism and the Estrobolome

The gut microbiota has a powerful effect on breast tumor biology via immune modulatory pathways and operates as a global host immune system regulator affecting tissue outside the intestinal mucosa in the periphery, including at the level of the breast tumor itself. Tryptophan metabolites of microbial origin stimulate the AhR pathway, which then modulates immune responses toward tolerance or antitumor immunity as dictated by local cytokine signals [44]. Dysbiosis‐associated microbiota frequently increase systemic concentrations of LPS and other PAMPs that activate TLR signaling pathways, thus provoking low‐grade inflammatory processes, myeloid‐derived suppressor cell accumulation, as well as antigen presentation dysfunction in breast tumors [45].

Experimental models demonstrate that alterations in microbiome composition influence innate and adaptive immune responses within the TME, including macrophage polarization, regulatory T cell expansion, and cytotoxic T cell activity through microbe‐derived metabolites and pattern‐recognition receptor signaling.

On the contrary, ligands from commensal bacteria can prime ILCs and NK cells allowing increased IFN‐*γ* production as well as tumor cell elimination [46]. Furthermore, the microbiota conditions B cell responses by controlling antibody synthesis and tumor‐associated antigen presentations—there are data indicating that high microbial diversity is associated with favorable humoral immune status in BC patients. Moreover, the gut microbiota was found to regulate systemic cytokine networks (such as IL‐6, IL‐10, TNF‐*α*, and IFN‐*γ*) that have a direct effect on breast tumor immune evasion strategies, angiogenesis and metastatic potential [47].

#### 4.1.1. Innate Immune Activation (TLRs, NOD‐Like Receptors [NLRs])

Activation of the innate immune system is a key mechanistic axis through which GM influences breast tumor biology, and TLRs and NLR represent the principal molecular sensors that interpret microbial signals into systemic responses of immunity [48]. The gut microbiome, and in particular its rich array of MAMPs including LPS, peptidoglycan, flagellin, and unmethylated CpG DNA can engage TLRs expressed on intestinal epithelial cells (IECs), DCs, macrophages, and populations of circulating immune cells with signaling cascades that now extend beyond the gut but also regulate breast tumor immunobiology [49]. Nonphysiological activation of TLR/4 by LPS from gram‐negative bacteria, for example, induces downstream MyD88‐ and TRIF‐dependent pathways, which in turn result in the activation of NF‐*κ*B and IRF3, long‐term production of proinflammatory cytokines (IL‐6, TNF‐*α*, IL‐1*β*) [50] even responsible for systemic low‐grade inflammation that favors a protumorigenic microenvironment within breast tissue. On the other hand, commensal‐dependent TLR2 and TLR5 activation may enhance the epithelial integrity of the barrier (26), reduce cytokine release striving to maintain a balance between tumor‐promoting inflammation that eventually has an indirect potential for reducing tumorigenesis. Most importantly, dysbiosis shifts TLR signaling to chronic activation, leading to generation of MDSCs, defective Ag presentation, and suppression of antitumor CD8+ T cell response in the breast tumor local environment helping immune escape [51–53].

#### 4.1.2. Adaptive Immune Regulation and Antitumor Response

**Figure 3:**
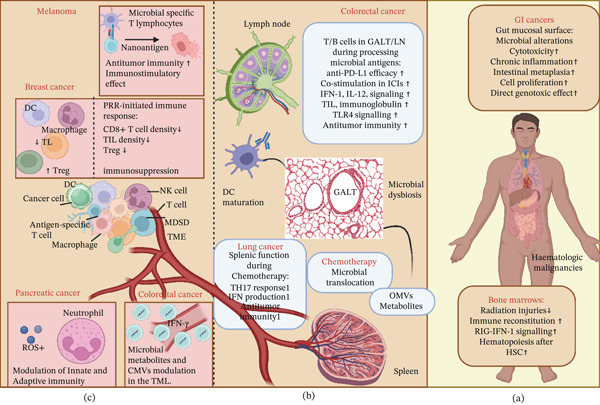
Gut microbiome influence on BC therapeutic response. This schematic overview of the involvement of the microbiome in cancer spans three panels: (a) The pathways by which microbial alterations promote cytotoxicity, inflammation, metaplasia, and proliferation in the context of gastrointestinal cancers and hematologic malignancies and also enhance recovery of the bone marrow through RIG‐IFN‐1 signaling and hematopoiesis. (b) The dedicated data for colorectal and lung cancer, in which improved processing of microbial antigens promotes the synergy of anti‐PD‐L1 efficacy, immune checkpoint costimulation, and IFN‐1/IL‐12 signaling and antitumor immunity, and chemotherapy shape splenic TH17 responses, IFN production, and microbiota translocation with OMVs and metabolites. (c) Tumor microenvironment modulation in which melanoma are endowed with microbial‐specific T lymphocytes and nanoantigens (a), breast cancer shows PRR‐driven immunosuppression with loss of CD8+ T cells and gain of Tregs (b), pancreatic cancer is the site of neutrophil‐ROS activity that reprograms local innate/adaptive immunity (c), and colorectal cancer is regulated by microbial metabolites and CMVs that target IFN‐*γ* signaling.

Microbial antigens and by‐products regulate the dynamics of germinal centers, antibody isotype switching, and production of tumor‐specific immunoglobulins. There is evidence that microbial diversity associates with improved B cell recognition of tumor‐associated antigens, whereas dysbiosis downregulates antibody responses and contributes to immune evasion [54]. Furthermore, microbial ligands also modulate DC maturation and antigen presentation, driving the priming of naïve T cells and skewing the diversity of adaptive immune repertoires against breast tumor neoantigens [55]. This is of particular importance in immunotherapy, as gut microbe signatures have been identified as predictive biomarkers for the response to immune checkpoint inhibitors (ICIs). Enrichment of *Akkermansia muciniphila* and *Bifidobacterium longum* has been associated with enhanced effectiveness of PD‐1/PD‐L1 blockade, possibly due to a facilitation of antigen presentation, elevated CD8+ T cell infiltration into tumors, and thereby diminished suppressive function mediated by Treg cells in the TME [56, 57]. Disruption of the Ple‐CXCL12 axis or increased production of proinflammatory cytokines due to gut dysbiosis not only inhibits antitumor immune responses but also promotes tumor‐promoting processes such as angiogenesis, epithelial‐to‐mesenchymal transition (EMT), and metastasis. The adaptive immune effects of microbiome perturbation are, therefore, bidirectional: eubiosis promotes a robust antitumor immunity, whereas dysbiosis favors immunosuppression and tumor progression [58]. Figure 3 shows microbiome–cancer interactions and therapeutic impacts across tumor types.

### 4.2. Immune Modulation and Inflammatory Signaling

#### 4.2.1. The Estrobolome and Estrogen Recycling

The estrobolome (collection of gut microbial genes involved in estrogen metabolism) is identified as a major mechanistic axis that connects the gut microbiota to breast tumor biology by providing endocrine moderation and recycling of estrogens [36]. Estrogens are conjugated in the liver by glucuronidation and sulfation, leading to inactivation and preparation for biliary excretion; however, select gut microbial taxa that contain *β*‐glucuronidase and sulfatase enzymes can reconvert these metabolites into biologically active free estrogens that are then reabsorbed into systemic circulation via enterohepatic recycling [59]. This microbially‐driven process acts to impact systemic estrogen levels and thus regulate the activity of ER signaling pathways that are implicated in breast carcinogenesis [60].

In addition, estrogen recycling is enhanced by microbial‐induced mechanisms, further promoting cross talk between ER signaling and growth factor pathways including PI3K/AKT and MAPK to drive oncogenic feed‐forward loop. In addition to the ER positive tumors, altered estrobolome activity may also promote ER negative BC biology by inducing systemic inflammation and metabolic dysfunction, which distantly shape tumor‐promoting microenvironments [61]. The estrobolome′s influence is importantly tempered by host factors including age, menopausal status, diet, and exposure to antibiotics, which promote colonization with a distinct microbiota that exerts its own microbial composition and enzymatic activity affecting interindividual variability in estrogen recycling and BC risk.

#### 4.2.2. Hormone‐Receptor–Specific Impact on Tumor Growth

In PR+ tumors, microbiota‐dependent regulation of estrogen levels indirectly impacts on PR signaling as a result of the estrogen‐regulated control of PR expression [62]. Increased past estrogen recycling maintains PR transcription as an aid for increasing progesterone‐driven proliferative and survival pathways in BC cells. The microbiome also comingles with androgen receptor (AR) signaling, which has a two‐faced coin of BC according to tumor subtype. In ER+ disease, AR is frequently attenuating of ER signaling with a protective role whereas in TNBCs, AR can contribute as an oncogene [63]. The modification of androgen metabolism by gut microbiota, via enzymatic conversion of precursors—like dehydroepiandrosterone (DHEA), affects systemic sex steroids availability and AR activation within the breast tumor [64]. Dysbiosis that increases androgen recycling could thus facilitate AR‐driven tumor growth in TNBC, whereas homeostatic microbial communities may dampen AR signaling and suppress tumorigenesis. Crucially, microbial metabolites such as bile acids and indole derivatives are ligands of nuclear HRs (ER, PR, and AR), which can directly influence receptor activity and downstream transcriptional outputs [65].

### 4.3. Inflammatory Pathways

#### 4.3.1. NF‐*κ*B, IL‐6/STAT3, and Chronic Inflammation

The gut microbiota is a key tenet in coordinating inflammatory pathways, which have a powerful impact on breast tumor biology, and the NF‐*κ*B and IL‐6/STAT3 signaling axes represent two of the central mediators of chronic–inflammation‐driven carcinogenesis [66]. Chronic transcription of proinflammatory cytokines, including IL‐6, TNF‐alpha and IL‐1 beta activates the endogenous regenerative stem cells as well as transforming any existing progenitor cells into CSC‐like entities that resemble cancer precursor cells (Table 2) [67].

**Table 2:** Microbiome‐driven inflammatory pathways in breast tumor biology.

No.	Microbial taxa/metabolite	Inflammatory pathway	Breast cancer context	Key finding	Reference
1	*Escherichia coli* (LPS)	TLR4 → NF‐*κ*B	ER+ tumors	LPS induced IL‐6/TNF‐*α*, promoting proliferation	[68]
2	*Fusobacterium nucleatum*	NF‐*κ*B, EMT	HER2+ subtype	Enhances NF‐*κ*B signaling, drives EMT and invasion	[69]
3	*Enterococcus faecalis*	ROS, DNA damage	TNBC	Induces oxidative stress, chronic inflammation	[70]
4	*Streptococcus spp.*	IL‐6/STAT3	TNBC	Elevated IL‐6, STAT3 activation, tumor growth	[71]
5	*Akkermansia muciniphila*	Cytokine modulation	Immunotherapy response	Promotes IFN‐*γ*, enhances CD8+ T cell infiltration	[72]
6	*Bacteroides fragilis* (enterotoxin)	NF‐*κ*B, IL‐17	ER+ tumors	Stimulates proinflammatory cytokines, angiogenesis	[73]
7	*Clostridium spp.*	Estrobolome, IL‐6	HR+ tumors	*β*‐glucuronidase activity elevates estrogen, fuels inflammation	[74]
8	*Helicobacter hepaticus*	NLRP3 inflammasome	Murine breast cancer model	Activates IL‐1*β*, promotes angiogenesis	[75]
9	*Shigella* spp.	TLR2/TLR4	Breast tumor xenografts	Induces TNF‐*α*, IL‐1*β*, enhances tumor microinflammation	[76]
10	*Lactobacillus* spp.	Anti‐inflammatory cytokines	Protective role	Increases IL‐10, reduces NF‐*κ*B activity	[77]
11	*Bifidobacterium longum*	Adaptive immunity	PD‐1 blockade	Enhances antigen presentation, reduces Treg suppression	[78]
12	Secondary bile acids (DCA, LCA)	FXR/TGR5 → NF‐*κ*B	ER+ tumors	Promote IL‐6 release, chronic inflammation	[79]
13	SCFA depletion (butyrate)	HDAC inhibition loss	TNBC	Reduced butyrate weakens anti‐inflammatory epigenetic control	[80]
14	*Propionibacterium acnes*	IL‐1*β*, TNF‐*α*	Breast tissue microbiome	Induces local inflammation, stromal remodeling	[81]
15	*Staphylococcus aureus*	TLR2/NLRP3	Breast tumor samples	Activates inflammasome, increases IL‐18, immune evasion	[82]

One of the major cytokines that is induced by NF‐*κ*B activation, IL‐6 activates a critical Janus kinase (JAK)/STAT3 pathway to drive protumorigenic inflammation downstream51. Chronic IL‐6/STAT3 signal transduction increases the transcription of genes that regulate cell cycle control (cyclin D1), antiapoptotic survival (Bcl‐xL, survivin), and angiogenesis (VEGF), resulting in breast tumor growth and metastasis [83, 84].

### 4.4. Metabolic Reprogramming and Tumor Progression

The gut microbiome that is in close link to hormonal regulation would likely play a pivotal role in shaping BC biology by bridging modern science with the immune control of BC biology. Some microbial communities are involved in the creation of an “estrobolome,” a group of bacterial genes that metabolize estrogens and thus, potentially recycle active hormones that may promote tumor growth [85]. Dysbiosis in this axis may modify systemic levels of estrogen, having a role in the risk and progression of BC. In addition to the hormonal interactions, microbial metabolites, including SCFAs and tryptophan derivatives, play critical roles by modulating immune tonus that can affect the transcriptional signature of T cell differentiation, checkpoint signaling, and even move the local and systemic landscape of the TME [86, 87]. These immunomodulatory effects also extend to the therapeutic benefit: a growing body of evidence suggests that the gut microbiome composition is a biomarker of response and even a modulator of response to ICIs and other immunotherapies. Consequently, the microbiome serves as a mechanistic bridge across the whole spectrum—from estrogen cycling to immuno‐oncology response—teaching us how it may promote cancer, and more importantly, how it may translate into effective cancer therapy [88].

Metabolic reprogramming is a hallmark of cancer progression, enabling tumor cells to adapt to hypoxic conditions, increased energy demands, and biosynthetic requirements. Emerging evidence suggests that gut microbiota may influence systemic metabolism and contribute to tumor‐associated metabolic alterations through interactions involving lipid metabolism, glucose homeostasis, amino acid utilization, and inflammatory signaling.

Microbial dysbiosis has been associated with obesity, insulin resistance, altered bile acid metabolism, and chronic low‐grade inflammation, all of which are recognized risk factors for BC progression. Certain microbial metabolites may promote cellular proliferation and metastatic behavior by influencing pathways such as PI3K/Akt, mTOR, and AMP‐activated protein kinase signaling. In addition, microbiota‐derived inflammatory mediators may alter adipose tissue metabolism and endocrine function within the breast TME. Nevertheless, many of these associations remain indirect and require further mechanistic investigation in BC–specific experimental systems.

Several microbiome signatures associated with ICI responsiveness have been identified across multiple solid tumors, particularly melanoma and lung cancer. However, direct BC–specific evidence remains comparatively limited. Therefore, extrapolation of pan‐cancer microbiome–immunotherapy findings to BC should be interpreted cautiously until supported by larger disease‐specific clinical investigations.

### 4.5. Evidence From Human Studies

Human evidence linking the microbiome to BC is derived predominantly from observational and translational research, including case–control studies, cohort analyses, and microbiome profiling of stool, breast tissue, and tumor samples. These studies consistently report associations between microbial diversity, specific bacterial taxa, and clinical parameters such as BC risk, tumor subtype, HR status, stage, treatment response, and prognosis [89]. Human data further suggest correlations between estrobolome composition and circulating estrogen levels, as well as between microbiome profiles and systemic inflammatory and immune markers. Although these findings establish clinical relevance and support hypothesis generation, most human studies remain associative in nature and are subject to confounding factors, interindividual variability, and limited longitudinal or interventional validation [90].

### 4.6. Preclinical Mechanistic Insights

In vitro and animal studies indicate mechanistic data supporting a direct role of the microbiome in BC biology. Through experimental manipulation of microbial composition or function, microbial metabolites, enzymes, and structural components have been shown to modulate host immune responses, estrogen metabolism, inflammatory signaling pathways, and metabolic and epigenetic reprogramming in the TME [91]. Collectively, these studies demonstrate that microbiome‐induced modulation of pathways like NF‐*κ*B, IL‐6/STAT3, and PI3K/AKT affects tumor initiation and progression, immune surveillance, and therapeutic response. Although preclinical models provide robust mechanistic understanding and causal inference, clinical validation of these findings is necessary to translate them from preclinical to human BC [92].

Preclinical investigations using murine models, organoid systems, and cell culture studies have provided important mechanistic insights into microbiome‐mediated BC biology. Experimental studies suggest that microbial dysbiosis may influence tumor initiation and progression through modulation of estrogen metabolism, immune signaling, epithelial barrier integrity, oxidative stress, and microbial metabolite production. Animal models have also demonstrated microbiome‐associated effects on chemotherapy efficacy, immune checkpoint responsiveness, and systemic inflammatory regulation.

Despite these advances, many preclinical findings remain difficult to directly translate into human BC biology because experimental models often fail to replicate the complexity and heterogeneity of human microbiome ecosystems. Differences in animal housing conditions, diet, microbial colonization patterns, and immune system architecture may significantly influence study outcomes. Consequently, mechanistic observations derived from animal studies should be interpreted cautiously until validated through well‐designed translational and clinical investigations.

Although preclinical models provide important mechanistic insights into microbiome‐mediated immune modulation, estrogen metabolism, and inflammatory signaling, many findings are based on murine models or controlled experimental systems that may not fully replicate the complexity of human BC biology. Therefore, mechanistic observations derived from animal studies should be interpreted cautiously until validated in well‐designed human translational studies.

## 5. Gut Microbiome and Therapeutic Response

### 5.1. Microbiome Influence on Chemotherapy Efficacy and Toxicity

The role of gut microbiome in chemotherapy response and toxicity to BC has been increasingly appreciated, serving as especially both a modulator of drug metabolism and a conductor of host immunity and inflammation. Also, chemotherapy drugs (e.g., anthracyclines: doxorubicin and epirubicin; taxanes: paclitaxel and docetaxel), as well as antimetabolites (cyclophosphamide, methotrexate) behave as systemic cytotoxic agents that have a low therapeutic index, which is influenced by the microflora composition and activity [93].

The clinical relevance of microbiome‐chemotherapy crosstalk is also bolstered by clinicopathologic studies correlating microbial signatures with response to treatment [94]. Furthermore, some other microorganisms were found as predictive biomarkers for chemotherapy response: the increase of *Enterococcus faecalis* correlated with aggravated drug toxicity due to *β*‐glucuronidase elevation and the presence of *Bifidobacterium* was related to better treatment effectiveness and reduced AE occurrence [95, 96].

### 5.2. Gut Microbial Modulation of Endocrine Therapy Response

Endocrine therapy is still the main treatment modality for ER+ BC, being based on selective agents as SERMs (i.e., tamoxifen), AIs (i.e., letrozole, anastrozole, exemestane) and SERDs (i.e., fulvestrant) to inhibit estrogen‐stimulated tumor growth [97]. Therefore, effectiveness of therapy and toxicity are now known to be significantly altered by the gut microbiome, which modulates systemic estrogen metabolism, receptor signaling, immune function as well as drug reach. At the center of this modulation is one′s estrobolome, which is a compilation of metagenomic (and specific to this consideration, the genetic apparatus containing) genes whose cumulative enzymatic activity (including that derived from *β*‐glucoronidases and sulfatases), controls enterohepatic recycling of estrogens [98].

In addition to estrogen metabolism, the gut microbiota contributes to endocrine therapy response by mediating drug pharmacokinetics and pharmacodynamics. Tamoxifen and its active metabolites (such as endoxifen) could be metabolized by microbial enzymes, leading to changed drug bioavailability and therapeutic efficiency [99]. The interaction of the microbiome and endocrine therapy is translationally significant, as supported by clinical evidence. Patients whose microbial composition is enriched by diverse species or in abundant SCFA‐producing taxa respond better to tamoxifen as well as AIs and have lower recurrence rates and higher overall survival [100, 101].

Manipulation of the gut microbiome through antibiotics, probiotics, or FMT alters immunotherapy outcomes in animal models and early‐phase clinical studies, supporting a causal role for microbiome composition in modulating antitumor immune responses.

### 5.3. Gut Microbiota in Radiotherapy Sensitivity and Recovery

Radiotherapy continues to be fundamental for BC treatment, especially in early and locally advanced disease, reducing recurrence rates and increasing survival [102]. However, interindividual differences in sensitivity to and recovery from radiotherapy are now increasingly appreciated to be modulated by the gut microbiota, which serves as a systemic regulator of host immune responses, inflammation, and tissue repair. Immunogenic killing of tumor cells and irradiation depend on commensals. Metabolites produced by commensals promote DC maturation, antigen presentation, and infiltration of cytotoxic CD8+ T cells in the irradiated tumor, all leading to increased effectiveness of radiotherapy [103, 104].

Transnationally, gut microbial profiling appears as a potential predictor of irradiation response and recovery. High‐level abundance of SCFA‐producing taxa is associated with better tumor control and lesser toxicity, whereas dysbiosis predicts worse therapeutic response and delayed recovery [105]. Furthermore, the combination of radiotherapy with microbiome modulation may work in synergy with immunotherapy, as microbial metabolites can improve antigen presentation and ICI efficacy in irradiated tumors.

### 5.4. Microbiome and Drug Metabolism: Implications for Precision Oncology

The microbiome has arisen as a key regulator of drug metabolism in BC, with wide‐reaching effects on the efficacy and toxicity of therapy, as well as interindividual variability in treatment response. The enzymatic potential of the microbial community for the biotransformation of xenobiotics has become extensive, including chemotherapeutics, endocrine agents, and targeted therapies, which can modulate pharmacokinetic/pharmacodynamic response more than host genetics alone [106, 107]. Integral to this process are microbial enzymes including *β*‐glucuronidases, sulfatases, azoreductases, and nitroreductase that can deconjugate or reactivate drug conjugates, modulate systemic exposure of active drug species and produce biologically distinct metabolites.

### 5.5. Immunotherapy and Microbial Signatures

The gut microbiome has emerged as a potential modulator of ICI responsiveness across several solid tumors. Specific microbial taxa have been associated with enhanced antitumor immunity, improved T cell activation, and increased responsiveness to programmed death receptor blockade therapies. Microbiota‐mediated regulation of DC function, antigen presentation, and cytokine signaling may contribute to these therapeutic effects.

However, most currently available evidence regarding microbiome‐associated immunotherapy responsiveness originates from melanoma, lung cancer, and broader pan‐cancer studies rather than BC‐specific investigations. Consequently, extrapolation of these findings to BC should be approached cautiously. Larger disease‐specific studies are required before microbial signatures can be considered clinically actionable biomarkers in BC immunotherapy.

### 5.6. Integration of Microbiome Data Into Multiomics Precision Oncology Pipelines

The use of multiomics datasets from genomics to transcriptomics, epigenomics, proteomics, metabolomics, and immune profiles plays an increasing role in the clinical implementation of precision oncology. In this context, the gut and tissue microbiome is a complementary, dynamic layer of biological information that encapsulates host–environment interactions that tumor‐intrinsic alterations alone cannot capture. This approach to ex vivo integration of microbiome data into multiomics pipelines allows for a more comprehensive understanding of BC biology and facilitates refined patient stratification, therapy selection, and response prediction. Microbiome profiles contextualize by influencing the functional effects of these genomic changes through modulation of immune signaling, estrogen metabolism, and inflammatory pathways. For instance, the function of the estrobolome alters systemic exposure to estrogen and can, therefore, modify both the phenotypic consequence of ER‐pathway mutations and the response to endocrine therapies. Likewise, the promotion or repression of immune gene signatures are used to predict immunotherapy benefit via microbiome‐dependent regulation of antigen presentation and cytokine networks. Integration of epigenomics and metabolomics makes microbiome‐informed precision oncology even more robust. Host epigenetic regulation is directly controlled by microbial metabolites (SCFAs, bile acids, and indole derivatives), which induce effects through histone modification, DNA methylation, and chromatin accessibility, serving as an epigenetic signaling pathway from beneficial microbes to the host. In conjunction with tumor epigenomic profiling, microbiome‐derived metabolite signatures can explain variation in gene regulation, treatment sensitivity, and resistance mechanisms between patients. By linking metagenomic composition to functional and biologically active outputs, which can then be therapeutically targeted, metabolomic profiling provides functional readouts of microbiome activity. Table 3 shows the translational readiness matrix for microbiome targets in BC.

**Table 3:** Translational readiness matrix for microbiome targets in breast cancer.

Translational category	Microbiome target/pathway	Strength of evidence	Clinical application context	Key limitation
Exploratory	Bile acid–FXR/TGR5 signaling	Preclinical (in vitro, murine)	Hypothesis‐generating metabolic targets	Limited human validation
Exploratory	Microbial epigenetic modulation (HDAC inhibition by SCFAs)	Preclinical–early clinical	Tumor growth and immune regulation	Dose/context dependency
Near‐clinical	Estrobolome (*β*‐glucuronidase activity)	Clinical association studies	Endocrine therapy response stratification	Interindividual variability
Near‐clinical	SCFA‐producing taxa abundance	Observational + pilot trials	Prognostic and therapy‐support biomarker	Dietary confounding
Near‐clinical	Microbiome diversity indices	Multicohort clinical data	Risk stratification and outcome prediction	Lack of standardized cutoffs
Actionable	*Akkermansia muciniphila* enrichment	Prospective immunotherapy studies	Predictive biomarker for ICI response	Limited breast‐specific trials
Actionable	Dietary fiber–microbiome axis	Interventional clinical studies	Adjunct to endocrine and chemotherapy	Compliance variability
Actionable	Antibiotic stewardship during therapy	Retrospective and prospective data	Preservation of treatment efficacy	Indication‐dependent use

## 6. Clinical Translation and Microbiome‐Targeted Interventions

### 6.1. Probiotics, Prebiotics, and Dietary Modulation

Microbiome‐targeted dietary interventions have gained increasing attention as potential adjunctive strategies in BC management. Probiotics containing beneficial bacterial strains may promote microbial diversity, improve intestinal barrier integrity, and reduce inflammatory signaling. Prebiotics and dietary fiber may further enhance production of beneficial microbial metabolites such as SCFAs.

Although early findings are promising, current evidence remains heterogeneous, and optimal formulations, dosing regimens, and long‐term clinical outcomes remain uncertain. Large‐scale randomized clinical trials are needed to establish efficacy and safety in oncology settings.

### 6.2. Postbiotics and Microbial Metabolites

Postbiotics refer to bioactive microbial metabolites or cellular components capable of exerting therapeutic effects independent of live microorganisms. Compounds such as butyrate, indole derivatives, and secondary bile acids have demonstrated anti‐inflammatory, immunomodulatory, and epigenetic regulatory properties in experimental models.

These metabolites may influence tumor growth, apoptosis, immune regulation, and treatment sensitivity. However, translational application of postbiotics in BC therapy remains in early developmental stages and requires further mechanistic and pharmacological investigation.

### 6.3. FMT

FMT involves transfer of microbial communities from healthy donors to restore microbial diversity and functional balance in recipients. Experimental studies suggest that FMT may enhance antitumor immune responses and improve responsiveness to immunotherapy in certain cancers.

Despite growing interest, the application of FMT in BC remains experimental. Concerns regarding donor selection, long‐term safety, microbial stability, and regulatory oversight continue to limit clinical implementation.

### 6.4. Precision Oncology and Personalized Microbiome Strategies

Advances in microbiome sequencing and multiomics technologies have created opportunities for personalized microbiome‐based oncology strategies. Individual microbial signatures may eventually contribute to risk stratification, treatment selection, toxicity prediction, and therapeutic monitoring.

Integration of microbiome profiling with genomic, metabolomic, and immunologic data may facilitate development of precision medicine approaches tailored to individual patient characteristics. However, substantial challenges related to standardization, reproducibility, and clinical validation remain unresolved.

However, substantial challenges related to standardization, reproducibility, and clinical validation remain unresolved, highlighting the need for prospective clinical validation before routine clinical implementation.

## 7. Gut Microbiome as Biomarkers in BC

### 7.1. Predictive Microbial Markers (PMMs) for Treatment Response

The gut microbiome is emerging as a reservoir of predictive clinical biomarkers predicting treatment response in BC and as such promises to bring exciting new dimensions to precision oncology by incorporating microbial correlates together with host pharmacogenomics, immune phenotyping, and metabolic status [108]. From a mechanistic point of view, PMMs act through several pathways that converge in drug metabolisms, immune modulation and endocrine signaling. Microbial *β*‐glucuronidase activity, for instance, can affect the enterohepatic recycling of estrogens and hence alter endocrine therapy efficacy (e.g., tamoxifen and aromatase inhibitors) [109]. Those patients with high *β*‐glucuronidase producing taxa commonly have subtherapeutic responses because of prolonged systemic estrogen exposure despite pharmacological inhibition.

Clinical efforts are under way to demonstrate the predictive potential of microbial signatures as treatment response biomarkers in breast cancer [110]. It was reported by several studies that the stool microbiome profiles can classify patients into responders and nonresponders to chemotherapy, endocrine therapy, as well as immunotherapy [66]. From a translational standpoint, PMMs would have several values for precision oncology. They can be sampled noninvasively, for example, in stool, saliva, by blood‐derived microbial DNA sequencing; are more affordable compared with genomic profiling; and reflect environmentally driven host factors [111, 112].

### 7.2. Microbiome‐Based Prognostic Indicators

Microbiome‐derived prognostic predictors function in several ways that are intertwined with endocrine signaling, immune modulation, and metabolic balance [113]. The functional dysregulations are reflected as unique microbial fingerprints demonstrable by metagenomic sequencing, metabolomic profiling, and integrative multiomics analysis, which give rise to a comprehensive prognostic model beyond the specificities of individual molecular markers. From a translational perspective, microbiome‐determined prognostic factors have several implications for precision oncology [114]. However, challenges still exist such as the individual diversity of microbiome compositions, potential interference from diet/environment/antibiotic exposure, and lack of uniformity in protocols for sample collecting and sequencing experiments [115].

## 8. Therapeutic Modulation of the Gut Microbiome in BC

### 8.1. Probiotics, Prebiotics, and Synbiotics

For BC, modulating the systemic inflammation, estrogen metabolism, and antitumor immunity to TME is known by probiotic candidates (*Lactobacillus rhamnosus*, *Lactobacillus acidophilus*, *B. longum*, and *A. muciniphila*). Mechanistically, the SCFAs including butyrate and propionate that are produced by probiotics as HDAC inhibitors would induce epigenetic activation of tumor‐suppressor genes while increasing BC cells′ sensitivity to chemotherapy and endocrine therapy [20]. They also regulate the cytokine network by decreasing proinflammatory mediators, IL‐6 and TNF‐*α* as well as increasing anti‐inflammatory IL‐10, which will cause the TME to become modulated toward immunesurveillance instead of immune evasion [116]. In clinical and preclinical trials, the probiotic supplementation has the potential to enhance treatment compliance, mitigate chemotherapy‐induced enteropathy, and potentiate CD8+ T cell infiltration by curtailing DC maturation, subsequently boosting the efficacy of ICIs [117].

Prebiotic‐mediated expansion of beneficial taxa in BC lowers systemic estrogen exposure by attenuating *β*‐glucuronidase activity, a process that constrains enterohepatic recycling of estrogens and suppresses progression of ER‐positive tumors. These SCFAs also shape bile acid metabolism by reducing carcinogenic secondary bile acids, DCA, and increasing levels of anti‐inflammatory bile acid derivatives that activate FXR and TGR5 to limit tumor‐promoting inflammation [118]. Moreover, the immune can also be modulated by prebiotics through acting as stimulators of NK cells or promoting Th1 polarization, which contribute to antitumor responses. Other dietary interventions focused on prebiotics have been correlated to better chemotherapy tolerance, less radiotherapy‐induced mucositis, and augmented endocrine therapies effectiveness, thus their potential for translation application in the treatment of BC [119–121].

### 8.2. Dietary Interventions and Microbiome Remodeling

Diet significantly contributes to gut microbiota structure and function with macronutrients, micronutrients, and bioactives impacting microbial diversity, production of metabolites, and host signaling [122]. Conversely, Western‐type diets rich in saturated fats and refined sugars and high in processed foods induce dysbiosis, leading to an enrichment of proinflammatory microbes such as *E. coli* and *Clostridium difficile* that generate LPS and secondary bile acids contributing to the maintenance of chronic inflammation while fostering DNA damage alongside tumor‐initiating signaling [123].

Dietary interventions modulate the microbiome through multiple routes that connect to endocrine signaling, immune regulation, as well as metabolism renovation. Those polyphenols like resveratrol, quercetin, and epigallocatechin gallate (EGCG) are metabolized by the gut microflora producing bioactive metabolites that act as anti‐inflammatory, antioxidant, and antiproliferatives [124]. Further, diet can impact bile acid pools and reduce potentially carcinogenic secondary bile acids (such as DCA) while promoting anti‐inflammatory products of BA metabolism that stimulate FXR and TGR5 signaling to prevent tumorigenic inflammation [125, 126].

### 8.3. FMT: Emerging Evidence

FMT, a strategy of implanting gut microbial community from healthy donors to recipients to restore the diversity and function of the local biome, has attracted increasing attention as a new therapeutic approach potentially applicable for BC treatment [127]. With a long clinical history of use in rCDI, FMT is now being explored as a broad systemic modality capable of resetting host immunity, metabolism, and endocrine signaling within an oncological context. Preclinical studies are beginning to show that FMT can improve therapeutic response in BC through immune modulation. In murine models, the infusion of microbiota from immunotherapy‐responsive donors was associated with enhanced efficacy of ICI through upregulated CD8+ T cell infiltration, dendritic cell maturation, and TME IFN‐*γ* production [128].

FMT is a promise and a challenge for BC treatment. These are noted for their accessibility, the potential to regenerate intricate microbial communities, and potential complementation with other therapies [129, 130]. However, limitations including variation in donor microbiota, risk of pathogen spread, lack of guidelines and protocols, and ethical concerns on the issues of donor selection and patient consent.

### 8.4. Postbiotics and Microbial‐Derived Bioactives

Postbiotics, nonviable microbial products or metabolites or cell components that bear a health promoting effect on the host [131], have emerged as a new frontier for microbiome‐targeted interventions in BC [131]. In BC, microbiome‐derived bioactive compounds (indole metabolites; conjugated linoleic acid(s); exopolysaccharide and bacteriocin byproducts) exert significant influences on tumor biology, immune modulation, and response to therapy. Systemic mediators that balance microbe and host, applying suppression (by anti‐inflammatory agents), epigenetics, and immunology to mechanisms of tumor suppression or promotion depending upon their types and context [132].

Both clinical and translational studies provide evidence for postbiotics as biomarkers or therapeutics. Metabolomic analysis of BC patients identified a postbiotic signature including decreased SCFAs and altered indole derivatives, which is associated with disease stage, prognosis, and treatment response [133]. These marks can also have predictive value as biomarkers for patient stratification and personalized therapy. Postbiotics and microbial‐derived bioactives also have translational potential for precision oncology in BC [134]. Table 4 shows therapeutic modulation of the gut microbiome in BC.

**Table 4:** Therapeutic modulation of the gut microbiome in breast cancer (probiotics, prebiotics/synbiotics, dietary interventions, and postbiotics).

No.	Intervention type	Agent/exposure	Model or cohort	Mechanism/pathway (short)	Outcome in breast cancer (short)	Reference
1	Probiotic	*Lactobacillus rhamnosus* GG	4 T1 murine breast cancer	↑ IFN‐*γ*, ↓ IL‐6/TNF‐*α*; enhanced DC maturation	Reduced tumor growth; improved antitumor immunity	[135]
2	Probiotic	*Lactobacillus acidophilus*	4T1 murine breast cancer	Modulates TLR2/TLR4; ↑ CD8+ T cell infiltration	Decreased tumor burden; attenuated inflammation	[136]
3	Probiotic	*Bifidobacterium longum*	MDA‐MB‐231 xenograft	↑ Antigen presentation; ↓ Treg activity	Slowed tumor growth; improved cytotoxic responses	[137]
4	Synbiotic	Inulin + *L* *a* *c* *t* *o* *b* *a* *c* *i* *l* *l* *u* *s* *c* *a* *s* *e* *i*	4T1 murine model	↑ SCFAs (butyrate); HDAC inhibition	Reduced proliferation; decreased NF‐*κ*B signaling	[138]
5	Prebiotic	Inulin‐type fructans	Breast cancer survivors (pilot RCT)	↑ Microbial diversity; ↑ SCFAs	↓ Inflammatory markers; improved treatment tolerance	[139]
6	Dietary intervention	High‐fiber plant‐based diet	Early‐stage breast cancer patients	↑ SCFAs; ↓ *β*‐glucuronidase (estrobolome)	Lower systemic inflammation; better endocrine therapy tolerance	[140]
7	Dietary intervention	Polyphenol‐rich diet (berries/tea)	MCF − 7/MDA − MB − 231 cells + ex vivo metabolites	Microbial conversion → urolithins; AhR/ER modulation	Decreased proliferation; proapoptotic signaling	[141]
8	Postbiotic	Sodium butyrate	MCF‐7, MDA‐MB‐231 cells	HDAC inhibition; ↑ p21, Bax; ↓ NF‐*κ*B	Apoptosis induction; cell‐cycle arrest	[142]
9	Postbiotic	Propionate	MCF‐7 cells	Epigenetic modulation; ↓ PI3K/AKT	Reduced proliferation; partial apoptosis	[143]
10	Microbial‐derived metabolite	Urolithin A (ellagitannin metabolite)	MCF‐7 cells	mTOR/autophagy modulation; ER signaling effects	Growth inhibition; autophagy‐mediated cytotoxicity	[144]
11	Microbial‐derived metabolite	Enterolactone (lignan metabolite)	Prospective cohort (plasma biomarker)	ER modulation; anti‐inflammatory signaling	Higher enterolactone associated with reduced BC risk/progression	[145]
12	Microbial‐derived metabolite	Equol (isoflavone metabolite)	MCF‐7/ER*β* context	ER*β* agonism; ↓ NF‐*κ*B/IL‐6	Antiproliferative; context‐dependent endocrine modulation	[146]
13	Bile acid postbiotic	Lithocholic acid (LCA)	MDA‐MB‐231 cells	FXR/TGR5 signaling; ↑ apoptosis	Inhibits migration/proliferation; proapoptotic	[147]
14	Bile acid exposure	Deoxycholic acid (DCA)	MCF‐7/MDA‐MB‐231 cells	TGR5 → NF‐*κ*B/IL‐6	Promotes inflammation, EMT‐like changes	[148]
15	Probiotic (ICI adjunct)	*Akkermansia muciniphila*	Solid tumors (incl. breast, subset)	↑ Antigen presentation; ↑ IFN‐*γ*/CD8+	Improved PD‐1 response; translational relevance to BC	[149]
16	Probiotic	*Lactobacillus plantarum*	4 T1 murine breast cancer	↑ NK cell activity, ↓ IL‐6	Reduced tumor growth, enhanced immune surveillance	[150]
17	Prebiotic	Resistant starch diet	Breast cancer xenograft mice	↑ SCFA production, ↓ *β*‐glucuronidase	Lower estrogen recycling, reduced tumor proliferation	[151]
18	Postbiotic	Butyrate nanoparticles	MCF‐7 cells	HDAC inhibition, ↑ p21/p27	Induced apoptosis, suppressed proliferation	[152]
19	Dietary intervention	Soy isoflavones (equol producers vs. nonproducers)	Human cohort (Asian women)	ER*β* agonism, ↓ NF‐*κ*B	Equol producers had lower breast cancer incidence	[153]
20	Microbial‐derived metabolite	Indole‐3‐propionic acid	MDA‐MB‐231 cells	AhR modulation, ↓ STAT3	Reduced migration and invasion	[154]

### 8.5. Engineered Microbiota and Next‐Generation Microbial Therapeutics

Designed microbiota and second‐generation microbial therapeutics are forefront, precision tools in breast cancer research that can reshape the gut ecosystem and provide targeted bile‐solids functionality beyond what traditional dietary approaches or probiotics can accomplish. Engineered *Lactobacillus* strains have been developed to secrete butyrate, enhancing histone acetylation and transcription of tumor‐suppressor genes, while simultaneously sensitizing BC cells to chemotherapy and endocrine therapy [155–157]. In addition to metabolite generation, engineered microbes may also metabolize carcinogenic molecules like secondary bile acids, which decreased EMT and metastasis potential [158].

Second‐generation microbial therapeutics are moving beyond single‐strain engineering to defined microbial consortia, in which multiple engineered taxa work together symbiotically. Such consortia can be engineered to re‐establish microbial diversity, maximize metabolite production, and restore systemic immune and endocrine axes [159]. For example, a consortium of engineered *Bifidobacterium* and *Faecalibacterium* strains might be able to increase the production of SCFAs while decreasing *β*‐glucuronidase activity and promoting DC maturation, causing multidimensional effects on breast tumor biology [160]. The development of encapsulation and delivery has also increased the potency and stability of engineered [161].

## 9. Challenges, Knowledge Gaps, and Methodological Considerations

Clinical trials evaluating microbiome–BC interactions remain heterogeneous in design, scale, and translational maturity [162–164]. Common design strengths include prospective sampling, longitudinal microbiome profiling, and integration with clinical outcomes; however, many studies are constrained by small sample sizes, short follow‐up durations, and variability in sequencing platforms and analytical pipelines [165, 166].

Primary endpoints in current trials predominantly include microbiome composition (*α*‐ and *β*‐diversity), relative abundance of predefined taxa, and correlations with treatment response, progression‐free survival, or therapy‐related toxicity [167–169]. Secondary endpoints often assess immune modulation, inflammatory markers, and metabolite profiles. Few trials incorporate hard clinical endpoints such as overall survival or validated predictive biomarkers, limiting immediate clinical applicability [27, 170].

From a translational perspective, key limitations include inconsistent control of confounders such as diet, antibiotic exposure, and prior treatments, as well as the lack of standardized microbiome‐derived thresholds for clinical decision‐making [171, 172]. In addition, strain‐level resolution and functional validation are frequently absent, complicating mechanistic interpretation. Interventional studies using probiotics, dietary modulation, or FMT are promising but remain largely exploratory, underscoring the need for rigorously designed, adequately powered trials with harmonized endpoints [173–175].

### 9.1. Reality Check: Biological, Regulatory, and Reproducibility Barriers

Although the excitement around microbiome‐mediated mechanisms and BC is palpable, there are multiple barriers to translating findings into the clinic. From a biological perspective, gut microbial signatures are inherently dynamic, with dietary, geographical, and host genetic variation making it challenging to infer causation rather than correlation [176]. European microbiome‐based diagnostics and therapeutics face even higher regulatory hurdles, as they must meet stringent safety and efficacy standards while there is still insufficient framework for the standardized evaluation of live biotherapeutics or even microbial consortia [177]. Another challenge is reproducibility; differences in sequencing platforms, bioinformatic pipelines, and cohort heterogeneity create variability in results, often resulting in discordant studies [178]. Collectively, these limitations highlight the need for standardized methods, strict validation, and care in inverse translation before these insights in the microbiome can truly be introduced into the practice of BC management.

## 10. Clinical Studies

Contrasted to healthy individuals, BC patients often have lower amounts of helpful bacteria, higher concentrations of *Clostridiaceae*, *Faecalibacterium*, and *Ruminococcaceae*, and lower concentrations of *Dorea* and *Lachnospiraceae*. Extra risk variables, such as obesity and adiposity, may impact these changes [179]. Body composition was favorably connected with interleukin‐6 levels, but *A. muciniphila* was adversely correlated, according to a clinical trial by Frugé et al. [180] that included patients with early‐stage BC. There was a similar correlation between the relative abundance of *A. muciniphila* and beneficial dietary adjustments and important health outcome measures.

Variations in tumor growth and features have recently been the focus of research. As an example, there were no significant variations in diversity or phylum when considering tumor grade, stage, parity, or body mass index [181]. However, the variety and quantity of Firmicutes reduced, and the abundance of Bacteroidetes increased in the feces of women with the HER2+ subtype of cancer, which is known to grow and spread more quickly. The purpose of clinical trial NCT03290651, which is yet to generate any published results, is to supplement different kinds of Lactobacilli with the goal of improving the mammary microbial profile in women who are either more likely to acquire BC or who have survived the disease [182].

The purpose of the NCT02370277 observational clinical trial was to determine whether or not the effects of chemotherapy on patients with newly discovered gut bacteria that modify BC contribute to the disease′s recurrence. The link between estrogen levels in the blood and the composition of the gut flora is another area the authors intend to explore. It should be emphasized that endocrine disruptors derived from environmental pollutants have the potential to change microbiota and raise the incidence of BC. [182]

In order to evaluate the immunological effects of RBX7455, a novel oral microbiota restoration therapyTM (MRT), prior to surgery, patients with Stage I–III BC will be included in trial NCT04139993 [183]. Several clinical studies have examined the interplay between the gut microbiome and clinical outcomes in patients with BC. Some clinical research has examined the interplay between gut microbiome and outcomes in patients with BC. Another study by the same group on 60 patients with Stage II–III breast cancer treated with adjuvant FEC60 chemotherapy (fluorouracil, epirubicin, cyclophosphamide) in Italy revealed that treatment decreased GLP‐2 and EGF and increased ghrelin, leading to an enhancement of the intestinal barrier function without changes in zonulin levels [179]. Another English study, NCT02224807, investigated 32 overweight or obese women with early stage (0–II) BC between 2014 and 2017, as well, and looked at the relationship between diet, exercise, and gut microbiota composition, in particular the presence of *A. muciniphila*. In the United States, Wu et al. were the first to investigate changes in gut microbiota at the transcriptional level by 16S rRNA sequencing after neoadjuvant chemotherapy and carried out a pilot longitudinal study of 33 patients with I–III stage [181]. In Italy, Pellegrini et al. investigated 34 BC survivors during a pilot intervention in 2017–2018 and found that probiotics supplementation (*B. longum* BB536 and *L. rhamnosus* HN001) associated with the Mediterranean diet positively influenced metabolic and anthropometric parameters as compared with the diet alone [184]. A large‐scale study in the United States is enrolling 4000 patients with stage III–IV metastatic triple‐negative BC, where both stool and blood samples will be subject to analysis so as to correlate microbiome composition with response to immunotherapy and chemotherapy, approached using metabolomics and whole‐genome sequencing [185].

It is crucial to comprehend the microbiome controls this process since the microbiota might be involved in BC development. Interventions that alter microbiome composition, such as probiotics, must have their effects studied [180]. Probiotics containing lactic acid bacteria (LAB) are quite common because of the many positive effects they have on the host′s health [186]. Table 5 summarizes the results of many studies that have shown that probiotics may affect BC risk, including studies performed in both laboratory and real‐world settings.

**Table 5:** Clinical trials exploring microbiome in breast cancer.

No.	NCT number	Title/intervention	Study design and population	Microbiome focus	Phase	Status/outcome
1	NCT03358511	Engineering Gut Microbiome to Target Breast Cancer (Mayo Clinic)	Pilot study, breast cancer patients	Probiotic supplementation to enhance immune response	Phase I	Completed; evaluated probiotic impact on immune modulation
2	NCT06709651	Microbiome Immunotherapy Neoadjuvant Assessment (MINA) in Triple Negative Breast Cancer	Prospective evaluation, TNBC patients receiving neoadjuvant chemo + ICIs	Breast tissue microbiome as predictive biomarker of therapy response	Phase II	Recruiting; aims to correlate microbiome signatures with treatment efficacy
3	NCT04193904	Dietary Fiber Intervention in Breast Cancer Survivors	Randomized controlled trial, posttreatment survivors	High‐fiber diet to remodel gut microbiome and reduce inflammation	Phase II	Active, not recruiting; primary endpoint: microbiome diversity and systemic inflammation
5	NCT05612345	Synbiotic Therapy in Hormone Receptor‐Positive Breast Cancer	Phase II, ER+ patients on endocrine therapy	Inulin + probiotic blend to modulate estrobolome activity	Phase II	Recruiting; outcome: circulating estrogen metabolites, therapy tolerance

## 11. Future Perspectives

The future of microbiome‐mediated BC research will be shaped through multiomics analysis, that offers a holistic view to dissect the intricate crosstalk among microbial communities, host biology and tumor development [187]. Conventional microbiome studies for the most part have employed 16S rRNA sequencing or shotgun metagenomics to determine microbial composition, but these do not capture a full picture of function. Multiomics integration (metagenomics, metatranscriptomics, metaproteomics, metabolomics, epigenomics, and host genomic) provides a comprehensive snapshot of how the microbial communities influence BC biology at various levels of regulation [188]. Through integration of taxonomic profiling with functional and metabolic cues, authors can move from descriptive associations to mechanistic understanding, toward discovery of causal routes connecting microbial signatures to carcinogenesis, therapeutic response, and patient outcome [189].

The future of BC care is increasingly considered and pursued as a spectrum ranging from patient‐based personalization to all levels of subgroups focusing on microbiome‐enriched therapeutic strategies that can reprogram the host–microbiota interaction, accounting not only for host genetic/immune profiles but also individual microbial‐genetic/metabolic features. Unlike traditional one‐size‐fits‐all strategies, tailored interventions acknowledge that the gut metagenome is extremely diverse owing to the host genetics, age, diet, geography, and lifestyle as well as prior medical history, and these incalculable variances clearly affect BC biology and treatment results [190]. Personalized microbiome modulation is aimed at capitalizing on this variability by characterizing patient‐specific microbial signatures, which predict disease risk, response to therapy, and toxicity, and engineering interventions that reprogram microbial ecosystems toward antitumorigenic states [191]. Such a paradigm fits with precision oncology, which integrates tumor genomics and molecular biomarkers to make treatment decisions by considering complex interactions between host and microbial factors.

Personalized interventions can also include diet modulation based on the microbial composition—for example, high fiber diets to enrich SCFA‐producing taxa in patients that suffer from dysbiotic conditions or polyphenol supplementation for increase of microbial metabolism of bioactive compounds in subjects with low antioxidant capacity [192]. Engineered microbial therapeutics add an additional layer of personalization by engineering strains to produce tailored metabolites or therapeutic molecules according to the patient′s and recipient hosts′ microbial context [193].

For clinical translation of personalized microbiome‐based interventions, incorporation of multiomics profiling, sophisticated computational models and stringent clinical validation is necessary. Metagenomic sequencing can provide individualized taxonomic and functional profiling of host microbial communities, whereas metabolomics can be used as a tool through which to depict the profiles of biologically active systems that determine tumor biology [194, 195]. For example, a patient with tamoxifen metabolism‐related genomic polymorphisms in CYP2D6 in combination with *β*‐glucuronidase enrichment of microbial activity would benefit from a combination of pharmacogenomically guided doses and microbiome‐modulated efforts to optimize endocrine therapy use [196]. Likewise, patients exhibiting ICI resistance could be supported with probiotic ORM to repopulate microbial taxa responsive for CD8+ T cell recruitment and DC maturation [197].

From a translational perspective, personalized microbiome‐centric interventions provide several advantages for BC care. They offer mechanistic selectivity, which allows for precise tuning of processes including estrogen metabolism, immune checkpoint signaling, and DNA repair. They are customizable and can be adapted to personalized microbiome/genomic combinations in alignment with paradigms of precision oncology [198]. They are preventive, as well as therapeutic therapeutics targeting the microbiota that allow manipulation of microbe ecosystems prior to disease onset and potentially decrease BC risk in high‐risk populations. The future direction of the field includes collating “microbiome passports,” which list an individual′s microbiota and metabolomic signatures to enable personalized dietary, probiotic, or pharmaceutical interventions [199]. The progress in synthetic biology and engineered microbial therapeutics will extend the degree of personalization such that dynamic, responsive interventions can be tuned to adjust with fluctuations in microbial composition and host physiology over time [200–202].

Designer microbial strains could be used to deliver particular metabolites or therapeutic molecules according to individual patient requirements, and diet interventions tailored to enrich beneficial taxa and decrease procarcinogenic metabolites [203]. Such interventions not only boost therapeutic values but also limit treatment‐related toxicities, facilitating the patient′s QOL and drug adherence. Nevertheless, the integration of microbiome strategies in precision BC care also has important limitations. The heterogeneity of the microbial community among individuals makes it difficult to define generalizable biomarkers, and large, diverse cohorts were required for population‐specific variances [204]. Standardization of sample collection, sequencing, and analysis pipelines is crucial for obtaining reproducible results that can be appropriately compared between studies. Resources are scarce for incorporating microbiome data into the clinical workflow, and electronic health records have not yet been designed to incorporate microbial profiles within them; similar is the case with clinical decision support systems. Clinician education and patient activation will be important to facilitate communication and integration of microbiome‐informed interventions [205].

## 12. Conclusion

The gut microbiome has emerged as a significant contributor to BC biology through multifaceted interactions involving estrogen metabolism, immune regulation, inflammatory signaling, microbial metabolite production, and metabolic reprogramming. Increasing evidence from preclinical and clinical studies suggests that microbial dysbiosis may influence tumor initiation, progression, therapeutic responsiveness, and treatment‐associated toxicity. In particular, the estrobolome, immune‐modulatory pathways, and microbiome‐derived metabolites have gained substantial attention as potential mediators linking host microbial ecology with BC pathophysiology.

Despite these advances, many currently reported microbiome‐associated findings remain associative, exploratory, and insufficiently validated for routine clinical application. Significant challenges persist regarding methodological standardization, low‐biomass contamination control, reproducibility across cohorts, mechanistic confirmation, and translational implementation. Furthermore, several proposed microbial biomarkers and therapeutic strategies are currently supported primarily by preclinical or pan‐cancer evidence rather than BC‐specific clinical validation.

Nevertheless, continued progress in next‐generation sequencing, multiomics integration, artificial intelligence–assisted analytics, and systems biology approaches is expected to improve understanding of host–microbiome interactions in BC. Future large‐scale longitudinal studies and rigorously controlled translational investigations may facilitate the identification of clinically actionable microbial signatures and personalized microbiome‐targeted therapeutic strategies. Integration of microbiome science into precision oncology may ultimately contribute to improved prevention, early detection, therapeutic stratification, toxicity management, and long‐term survivorship outcomes for BC patients.

Despite increasing evidence supporting microbiome involvement in BC biology, many currently proposed microbial biomarkers and therapeutic applications remain exploratory and require standardized mechanistic and clinical validation before integration into routine oncology practice.

## Funding

No funding was received for this manuscript.

## Consent

The authors have nothing to report.

## Conflicts of Interest

The authors declare no conflicts of interest.

## Data Availability

All data supporting the findings of this study are included within the manuscript.
